# miRNA as a Prognostic Marker in Small Lung Cell Carcinoma

**DOI:** 10.3390/genes16121465

**Published:** 2025-12-08

**Authors:** Michał Bednarz, Aleksandra Osińska, Julia Durda, Milena Kędra, Michalina Boruch, Julia Gontarz, Alicja Petniak, Janusz Kocki, Paulina Gil-Kulik

**Affiliations:** 1 Student Scientific Society of Clinical Genetics, Medical University of Lublin, 11 Radziwillowska Str., 20-080 Lublin, Poland; michal.bednarz727@gmail.com (M.B.); osinskaa1@tlen.pl (A.O.); julkadurda1@gmail.com (J.D.); milenakedra@op.pl (M.K.); misia140702@gmail.com (M.B.); gontarz.julia@gmail.com (J.G.); 2 Department of Clinical Genetics, Medical University of Lublin, 11 Radziwillowska Str., 20-080 Lublin, Poland; janusz.kocki@umlub.pl (J.K.); pgil.poczt@vp.pl (P.G.-K.)

**Keywords:** small-cell lung carcinoma, SCLC, microRNA, miRNA, biomarker, chemoresistance, exosomes, PI3K/Akt, Hippo pathway, prognosis

## Abstract

Small-cell lung carcinoma (SCLC) is one of the most aggressive and therapeutically challenging malignancies. It is characterised by rapid progression, early metastasis and frequent relapse. Despite considerable advances in molecular oncology, effective biomarkers for prognosis and treatment response remain elusive. In this review, we summarise and discuss recent evidence on microRNAs (miRNAs) as central regulators of SCLC biology and their potential clinical applications. A narrative review of the literature was conducted. Search of PubMed and Scopus databases identified 14 miRNAs, including miR-7-5p, miR-22-3p, miR-134, miR-181b, miR-200b, miR-335, miR-335-5p, miR-495, miR-24-3p, miR-30a-5p, miR-30a-3p, miR-100, miR-1 and miR-494, which are linked to tumour progression, therapy resistance and metastasis. These molecules influence several signalling cascades, including PI3K/Akt, Hippo, TGF-β, PARP1-mediated DNA repair and autophagy. Their abnormal expression correlates with patient outcome and may enable plasma- or exosome-based non-invasive monitoring. In particular, strategies that restore or inhibit miRNA activity using mimics or antagomiRs show promise in improving drug sensitivity and complementing current treatment options. Overall, emerging evidence supports the integration of miRNA profiling into precision oncology for SCLC, with the aim of refining diagnosis, risk assessment and therapeutic decision-making.

## 1. Introduction

Small cell lung cancer (SCLC) is a high-grade neuroendocrine carcinoma that occurs predominantly in current or former smokers [1]. It represents one of the most aggressive forms of lung cancer, characterized by rapid growth, early dissemination, and poor prognosis [2]. Clinical staging relies on imaging and clinical assessment, yet the molecular mechanisms underlying its progression remain incompletely understood [3].

MicroRNAs (miRNAs), a class of small non-coding RNAs of 20–22 nucleotides, have emerged as critical post-transcriptional regulators of gene expression [4]. Their aberrant expression has been implicated in multiple cancer-related processes, including proliferation, apoptosis, migration, and resistance to therapy [4,5,6]. MiRNAs participate in the regulation of stem cell signaling pathways, and their dysregulation may disrupt cell cycle control, leading to oncogenic transformation [7,8]. Consequently, specific miRNA expression signatures are being intensively studied as diagnostic and prognostic biomarkers in numerous malignancies due to ease of acquiring mentioned biological material and cost effectiveness [6].

In the tumor microenvironment, miRNAs influence immune cell infiltration and function by modulating dendritic cells, M1 macrophages, and T-helper lymphocytes [9]. They may act as oncogenes or tumor suppressors, affecting cell growth, motility, angiogenesis, immune evasion, and therapeutic response [5,10,11]. Notably, many miRNAs are secreted into biological fluids within exosomes and microvesicles, where they remain stable and biologically active [12,13,14,15]. The presence of circulating miRNAs in extracellular vesicles (EVs) enables their potential use as non-invasive biomarkers reflecting tumor dynamics and treatment response [6,15,16].

MiRNAs are frequently located in genomic regions prone to alterations, such as fragile sites, loss-of-heterozygosity (LOH) regions, and amplification zones. Through regulation of oncogenes, tumor suppressors, glycolytic enzymes, and transcription factors, they play a fundamental role in tumor metabolism and progression [17]. Given their stability and specificity, miRNAs have drawn growing attention as potential prognostic and therapeutic tools in SCLC, a malignancy still lacking effective molecular markers.

MiRNAs exhibit a distinct and unique expression pattern that differentiates SCLC from NSCLC. Detailed profiling analysis has shown that NSCLC is characterized by elevated expression of miRNAs involved in cell cycle regulation and signaling pathways, whereas SCLC often exhibits a unique set of low-expressed miRNAs. These differences suggest the potential use of miRNAs as diagnostic biomarkers and therapeutic targets specific to each subtype. Analyses by Shafat et al. and Sromek et al., among others, have shown that NSCLC has a specific miRNA expression profile that differs from SCLC [18,19].

Previous literature reviews about miRNAs in SCLC focused mainly on identifying multiple studies about single miRNA type and presenting potential applications as biomarker or future treatment target. Lack of works summarizing multiple miRNAs led to creation of this narrative review [20].

The aim of this review is to identify valuable studies about expression of various miRNAs in SCLC and present how those molecules influence cellular pathways in cancer cells and potential clinical uses for this biological data.

The present review summarizes recent evidence on miRNA expression patterns and their roles in SCLC development, progression, and treatment resistance, with particular focus on their potential as prognostic biomarkers and predictive biomarkers of treatment.

## 2. Materials and Methods

A narrative review of the literature was conducted to identify recent studies evaluating miRNAs in the context of small-cell lung carcinoma (SCLC) treatment and prognosis. The search was performed across PubMed and Scopus databases without date restriction, focusing on original articles and reviews published in English. The following combinations of MeSH terms and Boolean operators were used: (microRNA OR miRNA) AND (treatment OR therapy OR therapeutics) AND (SCLC).

Additional filters excluded non-English and non-peer-reviewed publications. Reference lists of included papers were screened manually to identify relevant studies not captured by the database search.

Study selection followed the PICO (Population, Intervention, Comparison, Outcome) framework. The population consisted of patients diagnosed with SCLC. The intervention was defined as the assessment of miRNA expression levels in tumor tissue, plasma, or serum. The comparison involved correlation between miRNA levels and diagnostic or prognostic parameters of SCLC, including treatment response and survival. The primary outcome was the prognostic value of individual miRNAs in SCLC.

**Figure 1:**
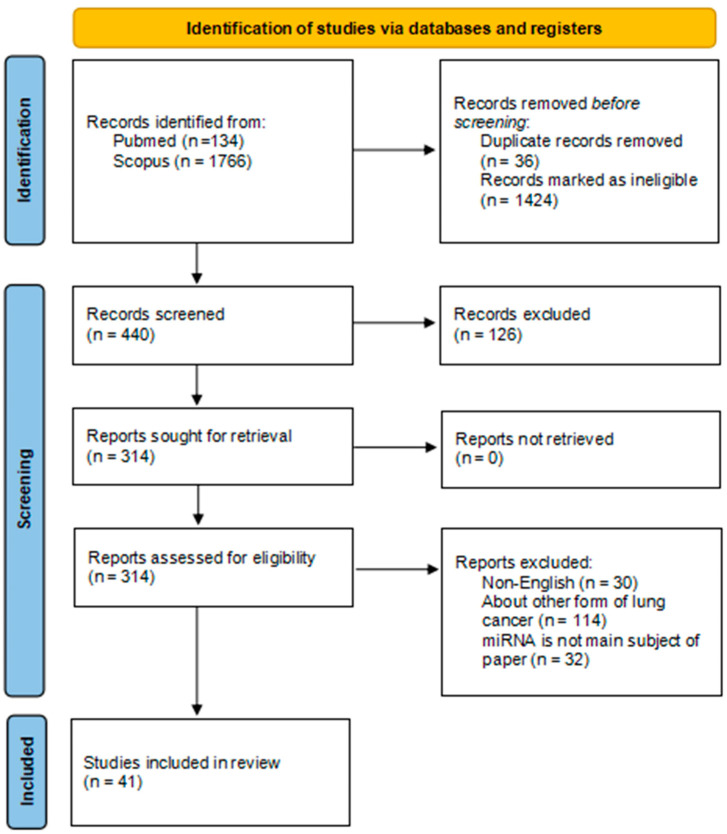
PRISMA flow diagram [21].

In initial search, 1900 papers were identified (Pubmed *n* = 134; Scopus *n* = 1766), from those 36 duplicate records were removed and 1424 were marked as ineligible, selected 440 records were manually screened and 126 works were excluded due to describing other form of cancer than lung cancer and/or due to only briefly explaining role of certain miRNA subtypes expression in SCLC. Remaining 314 works were screened, from those 30 non-English works, 114 works explaining role of miRNA expression in other forms of lung cancer (Non-Small Cell/Adenocarcinoma) and 32 works vaguely explaining role of certain miRNA subtypes expression in SCLC were excluded. Remaining 41 papers were included in the narrative review (Figure 1). The literature search primarily used the abbreviation ‘SCLC’. This approach was chosen to increase specificity and reduce the number of non-relevant NSCLC articles retrieved. However, relying exclusively on the abbreviation may have resulted in missing studies indexed under the full term ‘small-cell lung carcinoma’ or misclassified within electronic databases. This potential omission should be considered as a limitation of our review.

Abstracts and full texts were screened independently by the reviewers to determine relevance to the research aim. Studies were included if they provided quantitative or qualitative data on miRNA expression in SCLC patients and explored associations with biological behavior, chemoresistance, or clinical outcomes.

This review was based entirely on previously published research and did not involve new human or animal studies conducted by the authors. All data cited are derived from peer-reviewed scientific literature available in public databases.

## 3. Results

Based on the published literature review, 14 miRNAs: miR-494, miR-200b, miR-495, miR-100, miR-1, miR-7-5p, miR-335, miR-22-3p, miR-134, miR-335-5p, miR-24-3p, miR-30a-3p, miR-30a-5p and miR-181b with potential significance as biomarkers and therapeutic targets in SCLC were identified (Table 1, Figure 1).

## 4. Discussion

Small cell lung cancer (SCLC) accounts for approximately 15% of all lung cancer cases and remains one of the most aggressive and therapeutically challenging malignancies [1]. Despite recent treatment advancements due to clinical trials such as IMforte [44] and deLLphi [45], notable progress in molecular oncology, treatment outcomes for SCLC patients have improved only modestly in recent decades. The recurrent emergence of chemoresistance and the lack of validated molecular biomarkers continue to limit therapeutic success [3].

**Figure 2:**
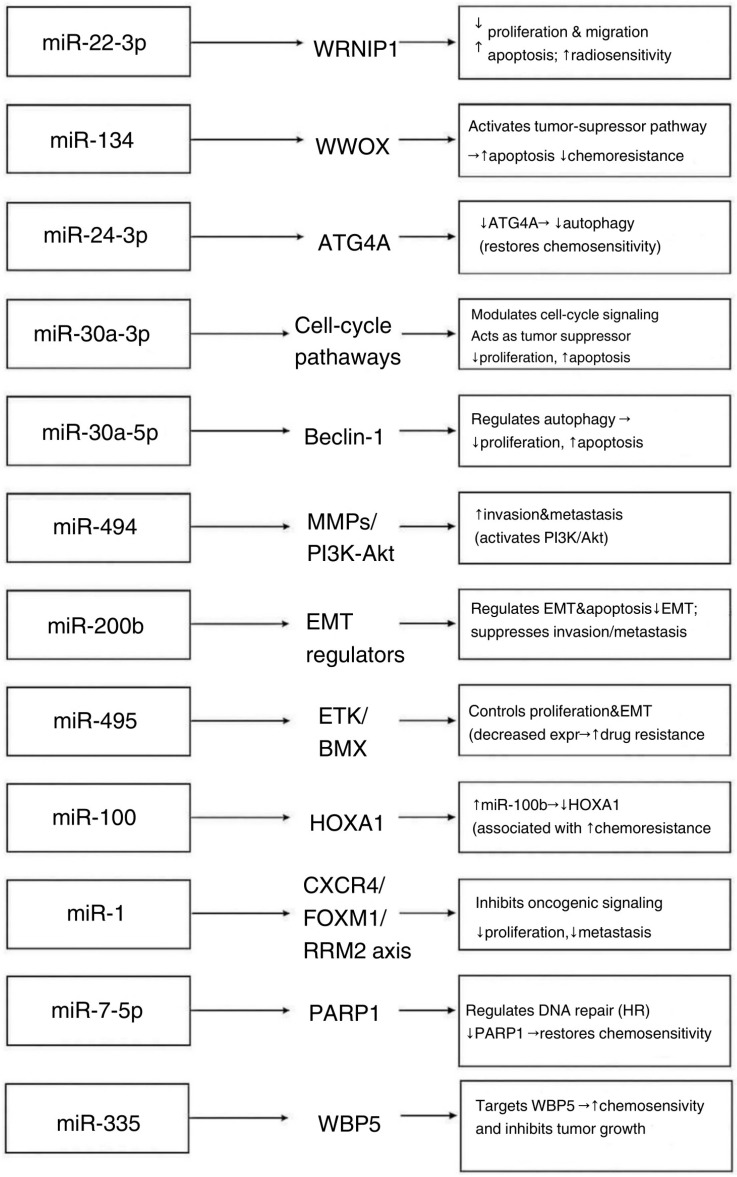
Effects of miRNA types expression on biological processes in SCLC cells [23,24,27,28,29,30,31,32,33,34,35,36,38,39,40,41,42,43]. Arrows indicate the regulatory direction, showing how each miRNA modulates its validated molecular target, which subsequently drives the described downstream biological effect in SCLC.

In small cell lung carcinoma (SCLC), genomic profiling has revealed significant chromosomal rearrangements and a high mutational burden, frequently leading to inactivation of tumor suppressor genes TP53 and RB1 [2]. This genetic heterogeneity has been associated with metastasis, treatment resistance, and tumor progression [2]. Because a single miRNA can regulate approximately 200 mRNA targets through imperfect base pairing, and mRNA expression profiles vary among cell types, a given miRNA may exert distinct effects in different cellular contexts [22] (Figure 2).

### 4.1. Predictive Markers of Treatment

#### 4.1.1. miR-7-5p

In SCLC cell lines, miR-7-5p was found to be downregulated in drug-resistant cells [31]. Its expression in doxorubicin-resistant cells was fourfold lower than in parental cells, as determined by quantitative RT-PCR [31]. MiR-7-5p directly targets the ADP-ribose polymerase 1 (PARP1) gene, causing its downregulation [32]. Overexpression of miR-7-5p prevented doxorubicin-induced homologous recombination (HR) by inhibiting *RAD51* and *BRCA1*, key HR repair factors [31]. This overexpression re-sensitized SCLC cells to doxorubicin [31]. These results suggest that restoring miR-7-5p levels could help overcome chemoresistance in SCLC, although the clinical utility of miR-7-5p as a biomarker has not yet been evaluated [31,32].

#### 4.1.2. miR-22-3p

MiR-22-3p, located on chromosome 17, was shown to be downregulated in SCLC cells, acting at least partly through regulation of the WRNIP1 gene [36]. One study reported that miR-22-3p expression in the NCI-H446 SCLC cell line was significantly lower than in normal lung cells [36]. Overexpression of miR-22-3p in these cells markedly increased apoptosis and inhibited migration and proliferation [36]. It also sensitized the cells to γ-irradiation [36]. These findings suggest that miR-22-3p functions as a tumor suppressor and a potential radiosensitizer or prognostic biomarker in SCLC, although its in vivo role remains to be confirmed [36].

#### 4.1.3. miR-200b

Members of the miR-200 family, including miR-200b, are key regulators of epithelial–mesenchymal transition (EMT) and apoptosis. In various cancers, reduced miR-200 expression has been associated with resistance to chemotherapeutic agents such as gemcitabine, cisplatin, and doxorubicin [26]. In SCLC specifically, miR-200b expression was found to be suppressed in chemoresistant tumors [27]. Concurrently, ZEB2—a transcription factor involved in TGF-β signaling and EMT—showed dysregulated levels in SCLC [25,26]. Positive ZEB2 protein expression in SCLC samples was linked to miR-200b dysregulation and multidrug resistance [27]. These data suggest that loss of miR-200b and consequent activation of ZEB2-mediated EMT may contribute to chemoresistance in SCLC [26,27,46].

#### 4.1.4. miR-495

In SCLC cell lines with acquired chemoresistance, miR-495 expression was found to be decreased while ETK/BMX kinase activity was increased [28]. Overexpression of miR-495 or knockdown of ETK/BMX suppressed cell proliferation and reversed drug resistance via inhibition of epithelial–mesenchymal transition (EMT) [28]. Clinically, low miR-495 or high ETK/BMX levels correlated with advanced tumor stage and shorter patient survival [28]. These observations suggest that restoring miR-495 or targeting ETK/BMX could provide a novel strategy to overcome chemoresistance in SCLC [28].

#### 4.1.5. miR-134

In SCLC, miR-134 is significantly downregulated, contributing to tumorigenesis and drug resistance [37]. Upregulation of miR-134 activates WW domain-containing oxidoreductase (WWOX), a tumor suppressor that promotes apoptosis and suppresses tumorigenicity [38]. Quantitative PCR analysis showed that miR-134 downregulation led to reduced WWOX activation and increased cell proliferation [38]. Conversely, higher miR-134 levels were associated with decreased expression of the drug transporter MRP1/ABCC1, thereby reducing chemoresistance and correlating with better patient outcomes [38].

#### 4.1.6. miR-335 and miR-335-5p

miR-335 has been identified as an important regulator of multidrug resistance (MDR) in SCLC [33]. Analysis of 62 SCLC biopsy specimens revealed that miR-335 directly targets the WW domain-binding protein 5 (WBP5) gene [33]. In multidrug-resistant SCLC cell lines, miR-335 was significantly downregulated compared with parental lines, whereas WBP5 expression was conversely increased [33]. WBP5 promotes MDR by enhancing cell proliferation and migration and by inhibiting apoptosis, and its high expression has been linked to shorter survival in SCLC patients. Overexpression of miR-335 in these models suppressed WBP5 expression, which in turn reduced tumor growth in vivo and inhibited proliferation while inducing apoptosis in vitro, thereby increasing chemosensitivity to cytotoxic drugs [33,34]. Mechanistically, WBP5 mediates drug resistance through the Hippo signaling pathway (WBP5–ABL–MST2–YAP1); WBP5 overexpression reduces phosphorylation of MST2 and YAP1, leading to nuclear accumulation of YAP1 and transcription of survival genes [33]. Thus, restoring miR-335 may counteract WBP5-driven chemoresistance, although its clinical utility as a biomarker remains to be determined [33].

Although miR-335-5p originates from the same precursor as miR-335, it represents a distinct mature strand with partially different regulatory targets in SCLC. Downregulation of miR-335-5p has been implicated in SCLC chemoresistance and metastasis [34,35]. Reduced miR-335-5p levels lead to upregulation of PARP1, contributing to cisplatin and radiotherapy resistance [34]. In addition, low miR-335-5p expression levels are associated with increased migration and bone metastasis through deregulation of IGF-IR and RANKL [35]. High WBP5 expression, observed in late-stage SCLC, has been associated with multidrug resistance and shorter patient survival [33,35], suggesting that reduced miR-335-5p expression may contribute to this phenotype. Restoration of miR-335-5p expression has been shown to suppress metastatic behaviors and enhance sensitivity to cytotoxic therapies [35].

#### 4.1.7. miR-24-3p

MiR-24-3p has emerged as a key regulator of lung cancer pathogenesis and chemoresistance [39]. In SCLC, reduced miR-24-3p expression correlated with resistance to etoposide (VP16) and cisplatin in combination therapy models [39]. Overexpression of miR-24-3p increased cell sensitivity to VP16–cisplatin treatment by inhibiting autophagy: it directly targets the autophagy gene ATG4A, and miR-24-3p introduction led to reduced ATG4A protein levels [39]. This reduction in autophagy contributed to the sensitization of SCLC cells to chemotherapy. In addition, miR-24-3p regulates the lncRNA SOX21 antisense RNA 1 (SOX21-AS1) and the oncogene PIM2 in SCLC cells [40]. High SOX21-AS1 expression in SCLC tissues was associated with low miR-24-3p activity; silencing SOX21-AS1 reduced proliferation and migration and increased apoptosis, effects that were reversed by miR-24-3p inhibition [40]. PIM2 was identified as a direct target of miR-24-3p: miR-24-3p overexpression decreased PIM2 levels, thereby inhibiting cell proliferation and promoting apoptosis [40]. These findings highlight the multifaceted role of miR-24-3p in SCLC chemoresistance and suggest it as a potential therapeutic target [39,40].

#### 4.1.8. miR-30a-5p

miR-30a-5p, a miRNA of approximately 22 nucleotides in length, acts as a tumor suppressor in several malignancies [42]. In SCLC, low miR-30a-5p expression enhances chemoresistance by upregulating Beclin-1, a key regulator of autophagy [42]. Overexpression of miR-30a-5p suppresses Beclin-1 expression, thereby inhibiting autophagy and restoring chemosensitivity to cisplatin [42]. These findings highlight the miR-30a-5p/Beclin-1 axis as a critical determinant of autophagy-mediated drug resistance in SCLC [42].

#### 4.1.9. miR-181b

In SCLC patients, circulating miR-181b levels were significantly lower than in healthy individuals [43]. During first-line chemotherapy, miR-181b expression progressively increased over the initial treatment cycles but remained below the levels observed in healthy controls [43]. These treatment-related dynamics suggest that miR-181b expression may reflect chemotherapy response in SCLC. Functionally, reduced miR-181b promotes proliferation, migration, and cisplatin resistance through upregulation of its direct target ACE2, whereas restoration of miR-181b suppresses ACE2 and enhances chemosensitivity [43].

#### 4.1.10. miR-100

miR-100 has been implicated in SCLC chemoresistance [29]. In resistant cells, miR-100 is markedly upregulated, resulting in reduced expression of HOXA1 [29]. Low HOXA1 expression is associated with poorer prognosis in SCLC (*p* < 0.05 by Fisher’s Exact Test) and shorter overall survival (*p* < 0.001 by the Kaplan–Meier method) [29]. These findings highlight the importance of the miR-100/HOXA1 axis in modulating chemoresistance [29]. Mechanistically, miR-100 directly targets the 3′UTR of HOXA1, and its upregulation suppresses HOXA1 expression, thereby enhancing chemoresistance [29].

### 4.2. Prognostic miRNAs

#### 4.2.1. miR-1

MiR-1 was found to be downregulated in tumor tissue and serum from SCLC patients [30]. Reintroduction of miR-1 into SCLC cell lines inhibited cell growth and metastasis [30]. Mechanistically, miR-1 directly targets the chemokine receptor CXCR4, which prevents the transcription factor FOXM1 from activating its target gene RRM2 [30]. This results in reduced oncogenic signaling, proliferation, and metastatic potential [30]. These data indicate that miR-1 acts as a tumor suppressor in SCLC [30].

#### 4.2.2. miR-30a-3p

miR-30-3p was found to be significantly downregulated in SCLC tissues [41]. In examined SCLC cells, levels of downstream neighbor of SON (DONSON) mRNA and protein were significantly reduced by miR-30a-3p transfection [27]. In apoptosis assays, DONSON knockdown increased the percentage of apoptotic cells in SCLC cell lines [41]. Results suggested that miR-30a-3p acts as tumor suppressor [41].

#### 4.2.3. miR-494

MiR-494 has been reported to have complex roles in SCLC. It can suppress cell proliferation by inhibiting both pro-apoptotic and anti-apoptotic factors [23]. However, miR-494 upregulation also promotes invasion: it increases matrix metalloproteinase (MMP) levels and activates PI3K/Akt signaling by targeting PTEN [23]. TGF-β1-induced miR-494 expression contributes to tumor invasion via upregulation of MMPs [23]. Activation of this axis triggers PI3K/Akt signaling and contributes to SCLC advancement [24].

Current knowledge of miRNA function in small-cell lung cancer (SCLC) is limited to a relatively small number of studies conducted in cohorts ranging from 3 to 86 individuals, and many of these are based primarily on in vitro or preclinical models. Further large-scale clinical studies are needed to validate specific miRNAs as reliable biomarkers and to determine their predictive value in real-world therapeutic settings. Integrating high-throughput sequencing data with functional assays will help define the miRNA–mRNA regulatory networks that drive tumour progression and treatment failure [2,17]. Ultimately, a deeper understanding of miRNA-dependent molecular mechanisms could lead to the development of new prognostic tools and targeted therapeutic approaches that may improve clinical outcomes in patients with SCLC [11]. Future research should also focus on exploring the potential of circulating and exosomal miRNAs as non-invasive biomarkers for early detection, disease monitoring, and assessment of therapeutic efficacy. The ability to measure miRNA levels in plasma or serum offers a promising avenue for dynamic evaluation of treatment response. Furthermore, the development of miRNA-based therapies—through synthetic mimics or antagomiRs—represents a novel strategy that could complement existing chemotherapy and immunotherapy regimens [5,10,12,13,14,16].

MiRNA profiling may be integrated into SCLC diagnostic and treatment protocols in the future, although challenges related to the development, delivery, toxicity, and specificity of miRNA-based therapies should be addressed in future studies [5,10,12,13,14,16]. Ongoing clinical trials, such as the TRIPLEX study [47] and “Small Extracellular Vesicle miRNAs as Predictive Biomarkers for Immunochemotherapy Efficacy in Extensive-stage Small Cell Lung Cancer” [48], are already exploring the role of miRNAs as biomarkers in SCLC.

Clinical implementation of miRNA-based biomarkers in SCLC requires careful consideration of analytical platforms and pre-analytical variables. qRT-PCR remains the most widely used technique due to its high sensitivity and relatively low resource requirements, but variability in normalization strategies and reference gene selection can impact the reproducibility of results across studies [5,10]. Microarray-based profiling enables parallel quantification of hundreds of miRNAs but offers lower sensitivity and a narrower dynamic range compared with sequencing-based methods [12,13,14].

High-throughput RNA sequencing provides the most comprehensive miRNA coverage, including the detection of novel miRNAs and isoforms, but is limited by higher costs, longer processing times, and the need for complex bioinformatic workflows [17]. Pre-analytical factors—including sample type (tissue, serum, exosomes), haemolysis, storage conditions, and RNA extraction method—introduce additional variability that complicates inter-study comparisons and the establishment of clinically relevant thresholds [5,12,13,14]. Harmonisation of pre-analytical protocols, analytical standards, and reporting guidelines will therefore be essential to ensure reproducibility and to enable reliable clinical translation of miRNA profiling in SCLC.

From a practical perspective, miRNA profiling in SCLC can be incorporated at several levels of clinical decision-making. Circulating and exosomal miRNA panels can support early detection and subtype differentiation alongside conventional imaging and histopathology, as suggested by plasma-based signatures described in lung cancer cohorts [1,12,13,16]. Furthermore, tissue- and blood-derived miRNA profiles could be used to improve prognostic stratification beyond standard clinical factors, for example by identifying patients at higher risk of rapid progression or early relapse [5,10,17]. Finally, miRNAs associated with chemoresistance, radiosensitivity and immune modulation—such as miR-7-5p, miR-335, miR-22-3p, miR-134 or miR-30a-5p—may serve as predictive markers to guide treatment intensity, the choice of systemic regimens or inclusion in clinical trials of novel agents [31,34,36,38,42]. In the longer term, dynamic monitoring of circulating miRNAs during therapy could enable real-time assessment of treatment response and emerging resistance [12,13,14,16].

However, several translational challenges need to be addressed before miRNA profiling can be integrated into routine treatment protocols for SCLC. Pre-analytical variability (sample type, processing time, haemolysis, storage conditions), differences in extraction and normalization methods, and heterogeneity of analytical platforms (qRT-PCR, microarrays, RNA-seq, ddPCR) currently limit study comparability and make it difficult to define robust cut-off points [5,10,17]. Furthermore, most available data come from small, single-centre cohorts with limited adjustment for confounding factors, and few multi-miRNA signatures have been prospectively validated in independent SCLC populations [1,2,13]. Large, well-designed prospective studies using harmonised protocols will therefore be essential to demonstrate that miRNA profiling provides additional prognostic or predictive value beyond current standards of care and to support its eventual clinical implementation in SCLC.

It should also be noted that the introduction of innovative miRNA-based therapies for SCLC, although promising, is associated with significant developmental, delivery-related and safety challenges. Effective therapeutic design requires chemical modifications that increase nuclease stability and reduce unintended interactions with non-target transcripts [5,10,49]. Another major barrier is efficient delivery. miRNA molecules are inherently unstable in the bloodstream, and their successful transport to SCLC cells requires specialised carriers such as lipid nanoparticles, polymer-based vectors or exosome-derived systems that protect the oligonucleotide cargo and enhance tumour uptake [12,13,14,49]. Therapy specificity represents an additional challenge. Because individual miRNAs regulate multiple genes, there is a substantial risk of off-target effects, systemic toxicity and activation of innate immune responses [5,16,49]. Therefore, the development of clinically relevant miRNA-based therapies requires coordinated optimisation of formulation strategies, preclinical validation in SCLC models and rigorous assessment of safety and biodistribution before their integration into therapeutic protocols can be considered.

The evidence summarised in this review is also subject to several methodological limitations that should be considered when interpreting the results. Many studies rely on small sample sizes, retrospective designs and heterogeneous inclusion criteria, which reduces comparability and increases the risk of selection bias. Only a minority of publications include multivariable analyses adjusted for confounders such as disease stage, prior treatment exposure, smoking status or tumour heterogeneity, meaning that observed associations between miRNA expression and prognosis or chemoresistance may be partially driven by baseline clinical differences rather than true biological effects. Technical variability in sample processing, RNA isolation, normalisation and assay platforms further contributes to inconsistency. In addition, functional studies often confirm only single predicted targets, whereas many miRNAs regulate multiple genes simultaneously, complicating the interpretation of causality. These limitations underscore the need for future large, prospective, clinically annotated cohorts, standardised laboratory procedures and harmonised reporting criteria to enable robust validation of miRNAs as biomarkers or therapeutic targets in SCLC.

## 5. Conclusions

The accumulating evidence summarized in this review highlights the pivotal role of miRNAs in regulating tumor behavior, including cell proliferation, apoptosis, migration, and multidrug resistance. Altered miRNA expression profiles influence numerous signaling pathways such as PI3K/Akt, Hippo, TGF-β, and autophagy-related networks. Several miRNAs—including miR-7-5p, miR-335, miR-22-3p, miR-134, miR-200b, miR-495, miR-30a-5p, miR-24-3p, miR-100, miR-1, and miR-494—emerge as promising candidates for diagnostic, prognostic, or predictive biomarkers. Their regulatory effects on key oncogenes (e.g., *SOX2*, *PARP1*, *WBP5*, *ETK/BMX*, *PIM2*, *IGF1R*) indicate that miRNA-based modulation could enhance therapeutic responsiveness and reduce resistance to standard chemotherapeutics such as cisplatin and etoposide [4,7,8].

Collectively, these findings underscore the potential of miRNA profiling as a valuable component of precision oncology in SCLC. Identifying robust miRNA signatures could contribute to early diagnosis, stratification of patients by risk, and individualized therapeutic strategies but more research is still needed to validate uses of miRNA in these applications.

## Figures and Tables

**Table 1:** Characterization of the identified miRNAs with potential significance as biomarkers and therapeutic targets in SCLC based on the literature review.

miRNA	Expression Trend in SCLC	Function in Normal Cells	Role as a Prognostic or Functional Marker in SCLC	Sample Type	Cohort Size	Endpoint	Signalling Cascades	Correlation with Patient Outcomes
miR-494	Upregulated	Not assessed	Promotes MMP expression, enhancing invasion and metastasis; activates the PI3K/Akt signaling pathway [22,23,24]	Cell lines (A549) [18] SCLC tissues, RNA-seq data of serum samples [24]	6 paired SCLC tissues and the RNA-seq data of serum samples from 36 SCLC patients and 118 healthy controls [24]	Proliferation, colony formation [22,24]	PI3K/Akt activation via PTEN suppression [23,24]; TGF-β1–driven MMP signalling [23]; circVAPA–IGF1R/AKT axis [24]	Promotes invasion and metastasis [23,24]; contributes to tumour progression [24]; no OS data reported
miR-200b	Downregulated	Regulates epithelial–mesenchymal transition (EMT) and apoptosis [25]	Reduced miR-200b promotes EMT; its overexpression suppresses invasion and metastasis [25]	Tissue samples [25]	65 SCLC tissues [25]	Multi-drug resistance via ZEB2 [25]	TGF-β/EMT via ZEB2 [25,26,27]	Association with EMT and multidrug resistance [27]; no survival correlations reported
miR-495	Downregulated	Controls proliferation and EMT-related signaling via ETK/BMX [28]	Decreased expression enhances drug resistance and correlates with advanced disease stage [28]	Clinical specimens + cell lines (NCI-H446 and NCI-H69) [28]	86 SCLC tissues and 60 normal lung tissues [28]	Chemoresistance via ETK/BMX [28]	ETK/BMX–EMT signalling axis [28]	Low miR-495 and high ETK/BMX correlate with advanced stage and shorter survival [28]
miR-100	Upregulated	Not assessed	Increased miR-100 expression suppresses HOXA1, contributing to chemoresistance; low HOXA1 correlates with poorer prognosis and shorter survival [29]	Clinical specimens + cell lines (H69AR and H69) [29]	63 SCLC tissue samples and 29 blood samples [29]	Chemoresistance via HOXA1 [29]	HOXA1 regulatory axis [29]	Low HOXA1 associated with worse prognosis and shorter OS (*p* < 0.001) [29]
miR-1	Downregulated	Not assessed	Inhibits oncogenic signaling and cell proliferation; suppresses metastasis by targeting CXCR4/FOXM1/RRM2 axis [30]	Tumor tissues + serum [30]	35 tumor samples and 8 serum samples [30]	Growth and metastasis suppression [30]	CXCR4/FOXM1/RRM2 axis [30]	Tumour-suppressive role; no clinical OS data reported [30]
miR-7-5p	Downregulated	Not assessed	Regulates doxorubicin-induced homologous recombination repair; restores chemosensitivity through PARP1 downregulation [31,32]	Cell lines [H69HR and H69] [32]	In vitro only [32]	Chemoresistance (doxorubicin) [32]	PARP1–RAD51–BRCA1 HR repair pathway [31,32]	Low miR-7-5p contributes to doxorubicin resistance [32]; no patient-based outcome data
miR-335	Downregulated	Not assessed	Targets WBP5; its downregulation leads to increased WBP5 expression, promoting multidrug resistance, enhanced proliferation and migration, and reduced apoptosis; restoration of miR-335 enhances chemosensitivity and inhibits tumor growth [33,34,35]	Cell lines (H69HR, H69, H446) [30,31] and 62 SCLC biopsy samples [33]	In vitro studies [30,31] 62 SCLC biopsy samples [33]	Chemosensitivity; multidrug resistance via WBP5/Hippo signaling [33,34,35]	WBP5–ABL–MST2–YAP1 (Hippo pathway) [33,34,35]	Low miR-335 → high WBP5 → MDR and shorter survival [33]
miR-22-3p	Downregulated	Not assessed	Suppresses proliferation and migration; induces apoptosis; increases radiosensitivity via WRNIP1 targeting [36]	Cell lines (BEAS-2B, NCI-H446and HEK293T) [36]	In vitro only [36]	Radiosensitivity [36]	WRNIP1-dependent DNA damage response [36]	Radiosensitizing effect; no clinical correlation data reported
miR-134	Downregulated	Activates WWOX tumor-suppressor pathway promoting apoptosis [37,38]	Reduces MRP1/ABCC1 expression, decreasing chemoresistance and improving outcomes [37,38]	Cell lines (H69) [38]	In vitro only [38]	Chemiresensitivity [38]	WWOX → ERK1/2 inhibition [38]; MRP1/ABCC1 regulation [38]	Reduced chemoresistance; improved drug response [38]
miR-335-5p	Downregulated	Expressed during embryogenesis and early cell differentiation; involved in regulation of DNA repair pathways [34,35]	Downregulation of miR-335-5p leads to upregulation of PARP1, contributing to impaired DNA repair, cisplatin resistance and reduced radiosensitivity; decreased miR-335-5p expression also enhances migration and bone metastasis via deregulation of IGF-IR and RANKL. Restoration of miR-335-5p expression suppresses these metastatic and drug-resistant phenotypes and improves sensitivity to cytotoxic therapy [33,34,35].	SCLC cell lines (H69/H69AR, H446) and tumor biopsy specimens [23,24,25,26,27,28,29,30,31,32,33,34,35]	62 SCLC biopsy samples [29] + in vitro studies [34,35]	Multidrug resistance (PARP1), radioresistance, migration and bone metastasis (IGF-IR/RANKL) [33,34,35]	PARP1 DNA repair [30]; IGF-IR/RANKL bone-metastasis signalling [31]; WBP5 axis [33,34,35]	Low levels linked to chemoresistance, radioresistance, migration, bone metastasis [33,34,35]
miR-24-3p	Downregulated	Modulates autophagy by targeting autophagy-related gene 4A (ATG4A) [39]	Suppresses ATG4A expression, thereby inhibiting autophagy and potentially restoring chemosensitivity in resistant SCLC cells [39]	Cell lines (H446/EP and H446) [39]	In vivo only [39]	Chemoresistance (cisplatin/etoposide combination (VP16–DDP) [39]	ATG4A-mediated autophagy [39]; SOX21-AS1/PIM2 axis [40]	VP16–cisplatin resistance mediated by ATG4A; proliferation/migration/apoptosis regulation [39,40]
miR-30a-3p	Downregulated	Modulates cell-cycle-related signaling pathways [41]	Acts as tumor suppressor; inhibits proliferation and induces apoptosis [41]	SCLC tissues + SCLC cell lines (SBC-3 and H82) [41]	3 clinical specimens of SCLC, 3 normal clinical specimens [41]	Proliferation, apoptosis [41]	DONSON-related cell-cycle pathway [41]	Tumour-suppressive; clinical data limited to *n* = 3 [41]
miR-30a-5p	Downregulated	Regulates autophagy via Beclin-1 [42]	Inhibits proliferation and induces apoptosis through Beclin-1-mediated pathway confirmed in SCLC and other lung cancer models [42]	SCLC tissues + SCLC cell lines (9H446/EP and Letp/EP) [42]	8 clinical specimens of SCLC, 4 normal clinical specimens [42]	Chemoresistance, apoptosis [42]	Beclin-1/autophagy pathway [42]	Cisplatin resistance driven by excessive autophagy [42]
miR-181b	Downregulated	Not assessed	Downregulation of miR-181b increases ACE2 expression, promoting SCLC cell proliferation, migration and cisplatin resistance; restoration of miR-181b suppresses ACE2 and enhances chemosensitivity [43]	SCLC cell lines (H446, H446/DDP) and serum samples from SCLC patients [43]	30 SCLC serum samples and 30 non-SCLC controls; functional assays in cell lines [43]	Chemoresistance mediated through ACE2 regulation [43]	ACE2 signalling axis [43]	Low miR-181b → cisplatin resistance, proliferation, migration; circulating levels change during chemotherapy [43]

## Data Availability

No new data were created or analyzed in this study. Data sharing is not applicable to this article.

## Abbreviations

The following abbreviations are used in this manuscript:

ABL	Abelson tyrosine kinase
ACE2	Angiotensin-converting enzyme 2
AKT	Protein kinase B
ATG4A	Autophagy-related gene 4A
BMX	Bone marrow tyrosine kinase gene on chromosome X (ETK/BMX)
CXCR4	C-X-C chemokine receptor type 4
DNA	Deoxyribonucleic acid
DONSON	Downstream neighbor of SON
EMT	Epithelial–mesenchymal transition
ERK1/2	Extracellular signal-regulated kinase 1/2
EVs	Extracellular vesicles
FOXM1	Forkhead box protein M1
HOXA1	Homeobox protein A1
HR	Homologous recombination
IGF1R	Insulin-like growth factor 1 receptor
lncRNA	Long non-coding RNA
LOH	Loss of heterozygosity
MDR	Multidrug resistance
MMP	Matrix metalloproteinase
mRNA	Messenger RNA
mTOR	Mechanistic target of rapamycin
PARP1	Poly (ADP-ribose) polymerase 1
PI3K	Phosphoinositide 3-kinase
PIM2	Proto-oncogene serine/threonine-protein kinase Pim-2
PTEN	Phosphatase and tensin homolog
qRT-PCR	Quantitative real-time polymerase chain reaction
RANKL	Receptor activator of nuclear factor kappa-B ligand
RB1	Retinoblastoma gene
RRM2	Ribonucleotide reductase regulatory subunit M2
SCLC	Small-cell lung carcinoma
SOX2	SRY-box transcription factor 2
SOX21-AS1	SOX21 antisense RNA 1
TGF-β	Transforming growth factor beta
TP53	Tumor protein p53
VP16	Etoposide (VP-16)
WBP5	WW domain-binding protein 5
WRNIP1	Werner helicase-interacting protein 1
WWOX	WW domain-containing oxidoreductase
YAP1	Yes-associated protein 1
ZEB2	Zinc finger E-box binding homeobox 2
